# Commentary: “Comparison of spike parameters from optically identified GABAergic and glutamatergic neurons in sparse cortical cultures”

**DOI:** 10.3389/fncel.2015.00157

**Published:** 2015-04-22

**Authors:** Andrea Becchetti, Enzo Wanke

**Affiliations:** Department of Biotechnology and Biosciences, University of Milano-BicoccaMilan, Italy

**Keywords:** action potential, Fano factor, GFP, MEA, multi-electrode array, neuronal cultures, spike width

Because of the increasing use of multi-site extracellular recording from neuronal networks, it would be extremely useful to determine reliable ways to distinguish with this method the main neuronal populations *in vivo* as well as *in vitro*. In a recent paper, Weir et al. (2015) studied the problem of correctly distinguishing the activity of GABAergic and glutamatergic neurons by multi-electrode array (MEA) recording, in primary neuronal cultures. Because we recently addressed the same problem, obtaining different results (Becchetti et al., 2012), we wish to discuss the main experimental differences between these papers. Discussing the possible reasons underlying the observed discrepancies should be instructive for the researchers attempting to establish culture conditions that approximate the *in vivo* situation.

In both studies, the GAD67-GFP knock-in mouse (Tamamaki et al., 2003) was used to unequivocally identify GABAergic cells. Waveform analysis led to the conclusion that spike width-based criteria are insufficient to reliably assign the recorded units to GABAergic or glutamatergic cells. However, Weir et al. (2015) did not observe significant electrophysiological differences between these neuronal populations, whereas Becchetti et al. (2012) found that some statistics systematically varied between inhibitory (i.e., GAD67+) and excitatory neurons, particularly the Fano factor (FF; the ratio between spike-count variance and mean).

Presumably, the discrepancy arises because of the very different experimental conditions applied in the two studies. In order to precisely attribute the spike waveforms to identified GFP+ or GFP− neurons, Weir et al. (2015) chose to plate the cells at the lowest density compatible with viability of neocortical cultures, which caused a low rate of network growth. Moreover, for MEA recording, cells were transferred to artificial cerebrospinal fluid, thus removing the cell-conditioned medium that is rich in regulating factors released by neurons as well as glial cells. According to our experience, these alterations cause not only a low-activity starting point, but also a long-lasting perturbation of the steady-state network activity. In fact, Weir et al. (2015) report an average GABAergic cell density of 115 neurons/mm^2^ to be compared with 434 in our conditions and the values reported for brain slices obtained from GAD67-GFP mice. For instance, cell counts approximately twice as high were measured in the inferior colliculus (Ono et al., 2005), a region in which the GABAergic cell density is probably close to the upper limit, at least in rodents' brain (e.g., Merchán et al., 2005). Considering that GABAergic cells constitute about 20% of the neurons (for GAD67-GFP mice, see Tamamaki et al., 2003), too low a neuronal density may impair a full network maturation, characterized by neurons displaying distinct firing modes. Moreover, both theoretical and experimental work underlines the importance of inhibition to explain the functional features of neocortical networks, which are thought to be characterized by balanced excitatory and inhibitory input (e.g., Haider et al., 2006; Wang, 2010). For these reasons, we chose to use higher cell densities, to maintain the density of inhibitory connections as close as possible to the physiological one.

A normal cell density allows a much faster growing of cultured networks from 2 to 20 days-*in vitro* (DIV). We could record synchronized activity as early as 2/3 DIV and 98% of electrodes were active at 7/8 DIV. Typical results obtained in these growing conditions are also shown in Gullo et al. (2014), in which Figure 1A reports the timestamp of 65 excitatory neurons (with average spike rate of 0.37 Hz) and 27 inhibitory neurons (with average spike rate of 1.44 Hz) at 12 DIV. In this case, the different firing modes are easily recognized by FF of, respectively, 3.97 ± 0.3 and 14.8 ± 0.9 (1 h recording, 8 s window). On the contrary, Weir et al. (2015) report ~10 and 13 active neurons/120 electrodes at 7/8 and 15 DIV, respectively, with spike rates of 0.18 and 0.48 Hz.

In conclusion, we believe that the different culture conditions applied in the two papers led to large differences in the activities of the reverberating neuronal networks, accompanied by different levels of maturation of the synaptic connections. These differences are likely to explain the differences in bursting statistics of the different neuronal types.

## Conflict of interest statement

The authors declare that the research was conducted in the absence of any commercial or financial relationships that could be construed as a potential conflict of interest.
